# THE STUDY OF WOMEN’S HEALTH ACROSS THE NATION (SWAN): FROM MIDLIFE ONWARD

**DOI:** 10.1093/geroni/igz038.1287

**Published:** 2019-11-08

**Authors:** Nancy E Avis, Nancy E Avis, Sybil Crawford, Alicia Colvin, Carol A Derby, Carrie Karvonen-Gutierrez, Maria M Brooks

**Affiliations:** 1 Wake Forest School of Medicine, Winston-Salem, North Carolina, United States; 2 Department of Social Sciences and Health Policy, Wake Forest School of Medicine, Winston-Salem, North Carolina, United States; 3 Graduate School of Nursing, University of Massachusetts Medical School, Worcester, Massachusetts, United States; 4 Department of Epidemiology, Graduate School of Public Health, University of Pittsburgh, Pittsburgh, Pennsylvania, United States; 5 Department of Epidemiology and Population Health, Albert Einstein College of Medicine, Bronx, New York, United States; 6 Department of Epidemiology, University of Michigan School of Public Health, Ann Arbor, Michigan, United States

## Abstract

The Study of Women’s Health Across the Nation (SWAN) is a multisite, multiracial/ethnic longitudinal study of women initially aged 42-52 (N=3302) designed to characterize the physiological and psychosocial changes that occur during the menopause transition and to assess their relations to subsequent health and age-related diseases. Each of seven clinical sites recruited non-Hispanic white women and women belonging to a predetermined racial/ethnic minority (African American, Hispanic, Chinese, or Japanese). Cohort eligibility was determined from a cross-sectional survey of 16,065 women in 1996-97 aged 40-55 who were aged 42-52, had an intact uterus and at least one ovary, and not using hormone therapy. Cohort participants have been assessed in-person approximately annually through follow-up visit 15 in 2017 using a standardized protocol of detailed questions about medical, reproductive and menstrual history; lifestyle and psychosocial factors; physical and psychological symptoms; and anthropometric measurements, reproductive hormones, bone and body composition, and cardiovascular health.

